# Urethral Injury Resulting in Hypovolemic Shock Following Traumatic Foley Catheter Removal

**DOI:** 10.51894/001c.144542

**Published:** 2025-09-30

**Authors:** Tara Knisely

**Affiliations:** 1 Department of Emergency Medicine Henry Ford Genesys

## 94

### INTRODUCTION

Traumatic Foley catheter removal is a rare but serious urologic emergency that can lead to complications such as urethral bleeding, penile and scrotal ecchymoses, bladder hematoma, and urethral injury.¹ Early recognition and prompt management are essential to prevent long-term morbidity.² Similar cases have reported severe anemia and bladder injury due to traumatic Foley removal.³,⁴

### CASE DESCRIPTION

We present the case of a 76-year-old male who arrived at the emergency department with acute urethral bleeding and significant scrotal ecchymoses and edema following the removal of a Foley catheter with the balloon intact. The patient reported immediate pain, spontaneous urethral bleeding, and hematuria following the incident. Examination revealed extensive scrotal and perineal ecchymoses and blood at the urethral meatus, without evidence of penile fracture. The patient was also hypotensive and tachycardic, raising concerns for hypovolemic shock secondary to acute blood loss. CT imaging of the pelvis demonstrated active urethral hemorrhage with diffuse soft tissue edema and bladder hematoma. The patient required resuscitation with blood products and vasopressor support. He ultimately underwent urgent operative management by urology with urethral fulguration, bladder hematoma evacuation and irrigation, and catheter reinsertion. He was closely monitored in the ICU by urology and was discharged home with Foley catheter in place after an uncomplicated hospital course.

### CONCLUSION

Traumatic Foley catheter removal can result in severe urethral and bladder injury, necessitating rapid assessment and intervention. Previous studies emphasize the importance of early imaging,⁵ timely surgical management when indicated,⁶ and vigilant post-procedural follow-up to prevent complications such as urethral stricture, infection, or ongoing hemorrhage.

